# The relationship between older adults’ self-management abilities, well-being and depression

**DOI:** 10.1007/s10433-012-0237-5

**Published:** 2012-07-04

**Authors:** J. M. Cramm, J. M. Hartgerink, P. L. de Vreede, T. J. Bakker, E. W. Steyerberg, J. P. Mackenbach, A. P. Nieboer

**Affiliations:** 1 Institute of Health Policy & Management (iBMG), Erasmus University, Burgemeester Oudlaan 50, 3000 DR Rotterdam, The Netherlands; 2 Department of Public Health, Erasmus University Medical Centre, Rotterdam, The Netherlands; 3 ARGOS Zorggroep, Schiedam, The Netherlands

**Keywords:** Abilities, Quality of life, Self-management, Well-being, Older adults

## Abstract

This study aimed to identify the relationship between self-management abilities, well-being and depression. Our study was conducted among older adults (>65 years of age) who were vulnerable to loss of function after hospital discharge. Three months after hospital admission, 296/456 patients (65 % response rate) were interviewed in their homes. The 30-item Self-Management Ability Scale was used to measure six self-management abilities: taking initiative, investing in resources for long-term benefits, taking care of a variety of resources, taking care of resource multifunctionality, being self-efficacious and having a positive frame of mind. Well-being was measured with the Social Production Function (SPF) Instrument for the Level of Well-being (SPF-IL) and Cantril’s ladder. The Geriatric Depression Scale was used to assess depression. Correlation analyses showed that all self-management abilities were strong indicators for well-being (*p* < 0.001 for all). Regression analyses revealed that investing in resources for long-term benefits, taking care of a variety of resources, taking care of resource multifunctionality and being self-efficacious were associated with well-being. While no significant relationship was found between well-being and having a positive frame of mind or taking initiative, regression analyses revealed that these self-management abilities were related to depression. Investing in resources for long-term benefits and taking care of a variety of resources were significantly related to depression. This research showed that self-management abilities are related to well-being and depression among older adults. In addition, this study identified key self-management abilities for older adults who had recently been discharged from a hospital.

## Introduction

Within the context of the growing older population and the threat of overloaded healthcare and welfare systems, older adults’ ability to take care of themselves for as long as possible has become increasingly important (Lewis 2003). It is acknowledged that successful ageing is not only a matter of having the right genes but also of the way in which individuals actively manage their ageing processes. Because successful ageing requires the proactive management of resources in an environment of increasing losses and declining gains (Baltes and Baltes 1990), it depends on individuals’ abilities to self-regulate or self-manage their lives and ageing processes.

Despite acknowledgement of the importance of individuals’ contributions to the process of successful ageing, and despite the existence of psychosocial theories of ‘successful ageing’ (Baltes and Baltes 1990; Brandtstädter and Rothermund 2002; Carstensen et al. 1999; Freund and Baltes 1998; Rowe and Kahn 1987, 1997; Schulz and Heckhausen 1996), relatively few suggestions have been made to help older adults achieve better and sustainable self-regulation of their well-being (Steverink et al. 2005). Self-regulation is often mainly related to the physical health aspects of ageing, such as treatment adherence, exercise and diet (Clark et al. 1992; Hopman-Rock and Westhoff 2000; Holman and Lorig 1997). Additionally, self-management interventions are usually focused on educating people about how to cope with specific age-related problems, such as depression (Moxon et al. 2001; Scogin et al. 1989), loneliness (Andersson 1984), increased risk of falling (Reinsch et al. 1992), insomnia (Morin et al. 1993) or chronic diseases (Glasgow et al. 2002; Lorig et al. 1984; Schreurs et al. 2003). The social and psychological aspects of life, however, such as social contacts, adaptation, and well-being, are also known to be important for successful ageing (von Faber et al. 2001). Many older adults suffer from a mixture of problems in multiple life domains, which makes them vulnerable to adverse outcomes, such as dependence on others, chronic diseases, and admission to an institution (Rockwood et al. 1994). Older adults who lack reserves in multiple life domains are likely to benefit more from a self-management intervention that addresses different kinds of age-related problems, rather than one that focuses on a single specific problem. Furthermore, many interventions address the problems of older adults who have already suffered substantial loss, such as those residing in nursing homes. An increasing number of studies, however, have suggested that the prevention of age-related decline may be more effective than the management of its consequences (He’bert 1997; Rockwood et al. 1994). For this reason, self-management interventions may best be aimed at older adults who are beginning to experience problems, rather than at those with serious problems that have already resulted in substantial loss.

In the literature, there are studies on life-management strategies (e.g. Freund and Baltes 2002), but studies investigating self-regulating abilities in relation to well-being are scarce (Schuurmans et al. 2005). According to the theory of successful self-management of ageing (Steverink et al., 2003), self-management abilities are the abilities people need for managing resources in such a way that sustainable positive well-being is reached (Frieswijk et al. 2006, p. 220). The self-management of abilities (SMA) theory (Steverink et al. 2005) offers concrete guidelines for the achievement of better self-regulation with regard to well-being. According to this theory, successful ageing is a life-span process of realising and sustaining well-being. Successful self-management of ageing thus concerns the ways in which people are able to realise well-being and how they can sustain it when their resources decline. The SMA theory distinguishes six self-management abilities: (1) having a positive frame of mind, (2) being self-efficacious, (3) taking initiative, (4) investing in resources for long-term benefits, (5) taking care of a variety of resources, and (6) taking care of resource multifunctionality. The first self-management ability ‘a positive frame of mind’ refers to the ability to adopt and maintain a positive frame of mind or positive expectations even after an illness event. The ability to have a positive frame of mind is expected to contribute to well-being because it extends the time-horizon and boosts confidence which, in turn, encourages people to engage in activities and not to give up easily (Steverink et al. 2005). The second self-management ability ‘being self-efficacious’ refers to the ability to gain and maintain a belief in personal competence to achieve well-being. The higher a person’s self-efficacy beliefs are, the more likely that person will, indeed, undertake the activities and efforts needed to maintain or improve well-being. The third self-management ability ‘taking the initiative’ refers to the ability to be instrumental or self-motivating to realise well-being. The fourth self-management ability ‘investment behaviour’ is important for achieving stability in resources and thus for the maintenance of well-being in the long run. Without investment behaviour there will be a (stronger) decline in resources and well-being (Steverink et al. 2005, 2006). The fifth self-management ability is the ability to gain and maintain a variety in resources. Variety here means having more than one resource or activity to achieve a specific aspect of well-being. For instance, having not only a partner for obtaining affection but also a close friend, means having more than one resource from which to obtain affection, which leads to a higher level of well-being (Nieboer and Lindenberg 2002). Multifunctional resources (sixth self-management ability) are those resources or activities that serve multiple aspects of well-being simultaneously and in a mutually reinforcing way. Because of this mutual reinforcement, multifunctional resources are of special importance for the realisation of well-being (Steverink et al. 2005; Nieboer et al. 1999, 2010; Nieboer and Lindenberg 2002). Based on the SPF theory (Lindenberg 1996), Steverink et al. (2005) argue that these six self-management abilities should be directed at the realisation of physical and social dimensions of well-being to achieve successful ageing. Physical well-being is realised when people obtain sufficient comfort (satisfaction of basic physical needs, such as food, drink and warmth) and stimulation (pleasant range of physical and mental activation) (Lindenberg 1996; Nieboer and Lindenberg 2002; Nieboer et al. 2005; Ormel 2002; Steverink et al. 1998). Social well-being is realised when people receive sufficient affection, behavioural confirmation and status, as forms of positive evaluations of what a person is (affection), does (behavioural confirmation) and has (status) (Lindenberg 1996; Nieboer and Lindenberg 2002; Nieboer et al. 2005). The SMA theory does not regard successful ageing as the process of neutralising losses and discrepancies, but focuses on individuals’ reserve capacities to realise and sustain physical and social well-being (Steverink et al. 2005). As a partly overlapping construct, general self-efficacy is related to self-management abilities, because self-management abilities also include aspects of self-efficacy. Other partly overlapping constructs are mastery and control beliefs. People with greater self-management abilities would feel more in control of their lives and are therefore expected to have a higher sense of mastery. The study of Schuurmans et al. (2005), however, showed that the concept of self-management abilities differs from that of self-efficacy and mastery, and that it contributes uniquely to the prediction of well-being.

There is a need for guidelines to enhance the self-management in ageing, not only to support older persons who have suffered specific losses but also to aid in the prevention and delay of ageing-related problems and to contribute to the (pro)active creation and maintenance of one’s own well-being. Individuals older than 65 years of age are more likely than those in other age groups to be admitted to acute care from the emergency department. Once admitted, they are at an increased risk for poor outcomes such as functional decline (Palmer 1998). Wu et al. (2000) found that one or more limitations developed within 2 months in 42 % of older patients with no baseline dependency at admission. Functional loss may lead to greater dependence, which results in a higher burden of care for informal caregivers (Covinsky et al. 1997, 1999, 2003), higher utilisation of professional health care and higher health care costs (Inouye et al. 1998). In addition, it may lead to renewed hospital admission, prolonged hospital stay and admission to a nursing home (Boyd et al. 2008; de Rooij et al. 2006). Therefore, it is important to prevent or reduce functional loss among elderly at an early stage. Self-management abilities may help older adults to maintain well-being outcomes who are at risk of function decline. Little, however, is known about the relationship between self-management abilities and well-being among older adults at risk of function loss. Only two empirical studies (Frieswijk et al. 2006; Schuurmans 2004) have shown improvement in overall self-management ability and well-being (vs. control groups) through the implementation of bibliotherapy and home-based training interventions. These improvements remained significant after 6 months for bibliotherapy (Frieswijk et al. 2006) and 4 months for individual home-based training (Schuurmans 2004). Schuurmans (2004) found that changes in self-management abilities and well-being were clinically relevant. Both interventions showed significant improvements in four of the six self-management abilities (self-efficacy, taking initiative, resource investment and resource variety), but not in positive frame of mind or resource multifunctionality. These studies, however, were conducted among frail elders in the community. We currently lack knowledge about the behavioural and cognitive processes underlying the realisation and maintenance of well-being among older adults recently discharged from the hospital. Because self-management interventions may best be aimed at older adults who are beginning to experience problems rather than at those whose problems have already resulted in substantial loss, it is necessary to identify self-management abilities that are important for well-being in this group. In addition to the relationship between self-management abilities and well-being, we also expect to find a relationship between self-management abilities and depression among older adults. For example, while a positive frame of mind was found to be related to well-being (Steverink et al. 2001), empirical evidence shows that a negative frame of mind is related to problems in adapting to widowhood (Nieboer 1997) and depression (Sarkisian et al. 2002). Moreover, depressive symptoms may occur when people’s capacity to produce well-being is lowered as a result of functional loss, or vice versa the ability to strive after important goals to realise well-being may be affected by depressive symptoms. Thus, this study aimed to identify the relationship between self-management abilities and well-being and depression among older adults who were vulnerable to loss of function due to hospitalisation.

## Methods

### Study population

Our research was based on a pilot study of older adults who had recently been admitted to a hospital. The results of the pilot study have been used to identify possible practical implementation problems in preparation for the main evaluation study and serve as a base for power calculations for the main study (Asmus-Szepesi et al. 2011). In the pilot study, all patients (>65 years of age), who were admitted to the Vlietland hospital between June 2010 and October 2010, were asked to participate, which led to the inclusion of 456 older patients at baseline (within 48 h after hospital admission). Three months after hospital admission, a total of 296 patients (65 % response rate) were interviewed in their homes. Exclusion reasons were: lost interest to participate (*n* = 52), too ill (*n* = 35), terminally ill (*n* = 5), objection by partner/family (*n* = 14), mentally not able (*n* = 8), private reasons (e.g. death of spouse; *n* = 4), questions not applicable (*n* = 8), no contact/unable to reach respondent (*n* = 12) and reason unknown (*n* = 22). Deceased patients were excluded from the study sample (*n* = 49). The study protocol was approved by the medical ethics committee of the Erasmus Medical Centre, Rotterdam, the Netherlands, under protocol number MEC2011-041. Informed consent was obtained from all participants.

Table 1 displays the characteristics of the study sample. The mean of respondents was 75.8 [standard deviation (SD) 6.8; range 65–94] years and just over half (54.2 %) were female. Just over half (56.6 %) of the respondents was married/living together and the others (43.4 %) were single, widowed or divorced. Most (55.9 %) of the respondents were living independently with others, about one-third (37.3 %) were living independently alone, and the others (6.8 %) were living in senior residences or nursing homes.

**Table 1:** Characteristics of the study population

Age [years; mean ± SD (range)]	75.8 ± 6.8 (65–94)
Gender (male)	45.8 %
Marital status (married/living together)	56.6 %
Education [mean ± SD (range)]	4.1 ± 1.6 (1–7)
Living situation	
Independently alone	37.3 %
Independently with other	55.9 %
Nursing home/senior residence	6.8 %
Self-management abilities [mean ± SD (range)]	
Taking initiative	2.7 ± 0.8 (0.2–4.8)
Investment behaviour	2.8 ± 0.9 (0.0–5.0)
Resource variety	2.7 ± 0.8 (0.4–4.6)
Resource multifunctionality	2.7 ± 0.9 (0.0–5.0)
Self-efficacy	3.2 ± 0.8 (0.4–5.0)
Positive frame of mind	3.2 ± 1.0 (0.0–5.0)
Well-being [mean ± SD (range)]	
Well-being (SPF-IL)	2.8 ± 0.4 (1.3–3.8)
Well-being (Cantril’s ladder)	7.4 ± 1.3 (0.0–10.0)
Depression (GDS)	6.2 ± 1.4 (3.0–12.0)

*SD* standard deviation, *SPF-IL* Social Production Function Instrument for the Level of Well-being, *GDS* Geriatric Depression Scale

### Measures

The original 30-item Self-Management Ability Scale (SMAS) consists of six five-item subscales, which is proven to be a viable and reliable instrument (Schuurmans et al. 2005; Cramm et al. 2012a, b). The 30 items were coded on a six-point scale with the categories ranging from ‘never’ to ‘very often’ (range 1–6). An example of the subscale taking initiative is: ‘how often do you make an effort to have friendly contacts with other people?’ An example of the subscale investment behaviour is: ‘do you ensure that you have enough interests on a regular basis (such as a hobby) to keep you active?’ Subscale resource variety: ‘do you have different occasions on which you have friendly contacts with others?’ Subscale multifunctionality: ‘the activities I enjoy, I do together with others’; subscale self-efficacy: ‘are you able to let others know that you care about them?’ And the subscale positive frame of mind: ‘When you have a bad day, how often do you think that things will be better tomorrow?’ The internal consistency of the overall scale was 0.90, and those of the subscales were taking initiative 0.73, investment behaviour 0.77, resource variety 0.68, resource multifunctionality 0.67, self-efficacy 0.78 and positive frame of mind 0.81. Higher scores indicate higher self-management abilities.

Well-being was measured with the 15-item version of the SPF Instrument for the Level of Well-being [SPF-IL(s)] (Nieboer et al. 2005). This instrument has been applied in a variety of studies, including successful ageing (Frieswijk et al. 2006). This scale measures levels of physical (comfort, stimulation) and social well-being (behavioural confirmation, affection, status). Examples of questions are: ‘Do people pay attention to you?’ (Affection), ‘Do you feel useful to others?’ (Behavioural confirmation), ‘Are you known for the things you have accomplished?’ (Status), ‘In the past few months have you felt physically comfortable?’ (Comfort), ‘Do you really enjoy your activities?’ (Stimulation). Answers could be given on a four-point scale, ranging from never (1) to always (4). A higher score indicates greater well-being. Cronbach’s alpha of the SPF-IL in our study was 0.72, indicating a reliable instrument.

Cantril’s (1965) ladder was used to assess satisfaction with life and reflects a general, cognitive evaluation of a person’s well-being. Respondents were asked to rate their lives in comparison to the best and worst possible lives they could imagine, on a scale of zero (worst possible life) to ten (best possible life).

We used the 15-item Geriatric Depression Scale (GDS-15) (Sheikh and Yesavage 1986) to evaluate depression. The GDS consists of a series of yes/no questions and is a widely used instrument to screen for depression in older individuals and medically ill populations. The sensitivity and specificity of the GDS-15 have been evaluated in a variety of older populations (Sheikh and Yesavage 1986), geriatric inpatients (Shah et al. 1996), primary-care outpatients (D’Ath et al. 1994; Lyness et al. 1997), older medical patients (Pomeroy et al. 2001) and subjects older than 85 years (de Craen et al. 2003). The optimal cut-off point for higher risk of depression is usually a score of five or more, a score of ten or more indicates depression (D’Ath et al. 1994). Cronbach’s alpha of the GDS-15 in our study was 0.78, indicating a reliable instrument.

Education was assessed on seven levels ranging from (1) no school or some primary education (6 years of education or less) to (7) university degree (18 years of education or more).

### Analysis

Descriptive analysis included the calculation of means and standard deviations. After calculating bivariate correlations, multiple regression analyses were performed to reveal significant associations of self-management abilities with well-being and depression after controlling for possible confounders age, marital status, gender and educational level. All statistical analyses were conducted with SPSS software (v.17.0; SPSS, Inc., Chicago, IL, USA).

## Results

The results of the descriptive analysis to characterise our study population are displayed in Table 1. Mean education level was 4.1 (SD 1.6; range 1–7) and the mean GDS score was 6.2 (SD 1.4; range 3.0–12.0), indicating that many of the respondents were depressed. The mean well-being score within our study population (2.8; SD 0.4; range 1.3–3.8) was comparable to that measured by Frieswijk et al. (2006) using the SPF-IL among slightly to moderately frail older adults (mean 2.8; SD 0.4).

Correlations of self-management abilities with well-being and depression are displayed in Table 2. These results indicate that all self-management abilities were strong indicators for well-being and depression (*p* ≤ 0.001 for all). Looking at the background characteristics only marital status was significantly correlated with well-being (measured with Cantril’s ladder) (results not shown).

**Table 2:** Correlations between self-management abilities and well-being

	Well-being (SPF-IL)	Well-being (Cantril’s ladder)	Depression (GDS)	1.	2.	3.	4.	5.
1. Taking initiative	0.46***	0.32***	−0.28***					
2. Investment behaviour	0.56***	0.41***	−0.30***	0.74***				
3. Resource variety	0.53***	0.43***	−0.27***	0.53***	0.59***			
4. Resource multifunctionality	0.53***	0.39***	−0.16***	0.44***	0.57***	0.58***		
5. Self-efficacy	0.59***	0.38***	−0.24***	0.64***	0.65***	0.53***	0.54***	
6. Positive frame of mind	0.30***	0.16***	−0.29***	0.32***	0.37***	0.21***	0.25***	0.54***

*SPF-IL* Social Production Function Instrument for the Level of Well-being, *GDS* Geriatric Depression Scale

*** *p* ≤ 0.001 (two-tailed)

Table 3 displays the results of the multiple regression analysis with well-being and depression as the dependent variables. Also in the multivariate regression analyses no significant associations were found between background characteristics, well-being and depression. The results indicate that investing in resources for long-term benefits (*β* = 0.18; *p* ≤ 0.001), taking care of a variety of resources (*β* = 0.21; *p* ≤ 0.001), taking care of resource multifunctionality (*β* = 0.18; *p* ≤ 0.001) and being self-efficacious (*β* = 0.30; *p* ≤ 0.001) were related to the SPF-IL measures of well-being. The regression analysis with the Cantril’s ladder measure of well-being revealed similar results: investing in resources for long-term benefits (*β* = 0.18; *p* ≤ 0.001), taking care of a variety of resources (*β* = 0.23; *p* ≤ 0.001), taking care of resource multifunctionality (*β* = 0.12; *p* ≤ 0.05) and being self-efficacious (*β* = 0.13; *p* ≤ 0.01) were significantly related to well-being. While no significant relationship was found between either measure of well-being (SPF-IL or Cantril’s ladder) and having a positive frame of mind or taking initiative, regression analyses revealed that these self-management abilities were associated with the GDS measure of depression. Taking initiative (*β* = −0.11; *p* ≤ 0.05), investing in resources for long-term benefits (*β* = -0.12; *p* ≤ 0.05), taking care of a variety of resources (*β* = −0.17; *p* ≤ 0.01) and having a positive frame of mind (*β* = −0.25; *p* ≤ 0.001) were significantly associated with depression. No significant relationship was found between depression and taking care of resource multifunctionality or being self-efficacious.

**Table 3:** Associations between self-management abilities and well-being, as assessed by multiple regression analyses (β, standardised)

	Well-being (SPF-IL)	Well-being (Cantril’s ladder)	Depression (GDS)
Age	0.10	0.09	−0.07
Married	−0.00	0.11	−0.04
Female	−0.06	−0.01	0.03
Educational level (1–7)	−0.01	−0.06	0.05
Taking initiative	−0.04	−0.04	−0.11*
Investment behaviour	0.18***	0.18***	−0.12*
Resource variety	0.21***	0.23***	−0.17**
Resource multifunctionality	0.18***	0.12*	−0.06
Self-efficacy	0.30***	0.13**	0.00
Positive frame of mind	0.00	−0.04	−0.25***
Adjusted *R* ^2^	44.6 %	23.6 %	19.8 %
*F*	23.460	9.652	7.927

*SPF-IL* Social Production Function Instrument for the Level of Well-being, *GDS* Geriatric Depression Scale

* *p* ≤ 0.05, ** *p* ≤ 0.01, *** *p* ≤ 0.001 (two-tailed)

## Discussion

This study aimed to identify the relationship between self-management abilities, well-being and depression among older adults who had recently been discharged from the hospital. As expected, our research showed that self-management abilities were significantly related to well-being and depression. In this population, well-being was significantly associated with investing in resources for long-term benefits, taking care of a variety of resources, taking care of resource multifunctionality and being self-efficacious; depression was significantly associated with having a positive frame of mind, taking initiative, investing in resources for long-term benefits and taking care of a variety of resources.

Ageing often implies declines in reserves and resources in multiple domains; these losses often reinforce one another (He’bert 1997; Lindenberg 1996; Nieboer and Lindenberg 2002; Schulz and Heckhausen 1996; Steverink et al. 1998; Steverink 2001). For example, the loss of social activities may negatively affect an individual’s mood, which, in turn, may reduce the energy available to take care of one’s physical health. This lack of energy may lead to further loss of social activities and resources, creating a cycle of mutually reinforcing declines (Steverink et al. 2005). Thus, a small loss in one domain may lead to downward spirals of resource loss in multiple domains (Lindenberg 1996; Nieboer and Lindenberg 2002; Nieboer et al. 2005; Steverink et al. 1998, 2005). Due to declining reserve capacities among older adults after hospitalisation that prevent them from compensating fully for certain resource losses, these individuals are at risk of decline in multiple domains and, ultimately, well-being. Thus, it may be especially important that recently hospitalised older adults have a diverse repertoire of self-management abilities that facilitates the disruption of potentially negative cycles in important domains of well-being. At the same time, this repertoire should include abilities to reinforce one’s strengths, thereby creating and consolidating important resources for the maintenance of well-being and the prevention of depression.

The limitations of this study should be considered when interpreting the findings. Most importantly, the data collected were cross sectional; as a result, causal relationships could not be inferred. While our study showed that self-management abilities and well-being are related, we did not investigate whether interventions aiming to enhance these abilities actually improved well-being. Further research is necessary to establish a causal relationship between self-management abilities and well-being, and to explore ways in which the self-management abilities of older adults can be improved. Additionally, based on earlier work by Steverink et al. (2005), we assumed that all self-management abilities play a role in older adults with functional loss, but we do not have any indication whether all six strategies are equally important. Furthermore, we did not investigate other partly overlapping constructs such as mastery and control beliefs. It is expected that people with greater SMA would feel more in control of their lives and therefore have a higher sense of mastery and self-control. We also did not include the role of functioning (e.g. cognitive, social, physical) on self-management abilities, which are also expected to influence self-management abilities (Cramm et al. 2012a). These should also be investigated in future research. Finally, many respondents were too ill to participate as was shown by attrition after hospital admission. The response rate of 65 % might seem low, and there is the potential danger of non-response bias, but it is much higher compared to other studies (Picavet 2001).

These findings are based on a pilot study that was conducted among older adults who had recently been admitted to a hospital in 2010 in the context of the Prevention and Reactivation Care Programme (Asmus-Szepesi et al. 2011). These older adults of the pilot study had not received care according to the Prevention and Reactivation Care Programme. This integrated care programme aims to support a multifaceted and multidisciplinary approach to senior care. The care is organised around several core components, including screening for vulnerability, early detection of health problems, multidisciplinary teamwork and case management. The main goal of the programme is to reduce the loss of function among older patients after hospital discharge. The results of this study suggest that older adults who had just been admitted to a hospital may benefit from self-management interventions that focus not only on a single specific (health) problem but also address all basic aspects of well-being, including those that help them to proactively maintain well-being, prevent depression and age successfully. Future research investigating self-management interventions in hospitals that address all aspects of well-being is necessary to prove its clinical value among hospitalised older adults.

## Conclusions

This research showed that self-management abilities are related to well-being and depression among older adults. In addition, this study identified key self-management abilities for older adults who had recently been discharged from a hospital. The successful self-management of resources was related to well-being, depression and the avoidance of or sufficient ability to cope with losses.
